# Diaphragmatic herniation after esophagogastric surgery: systematic review and meta-analysis

**DOI:** 10.1007/s00423-021-02214-9

**Published:** 2021-06-15

**Authors:** Davide Bona, Francesca Lombardo, Kazuhide Matsushima, Marta Cavalli, Valerio Panizzo, Paolo Mendogni, Gianluca Bonitta, Giampiero Campanelli, Alberto Aiolfi

**Affiliations:** 1 Division of General Surgery, Department of Biomedical Science for Health, Istituto Clinico Sant’Ambrogio, University of Milan, Via Luigi Giuseppe Faravelli, 16, 20149 Milan, Italy; 2 Division of Acute Care Surgery, LAC+USC Medical Center, University of Southern California, 2051 Marengo Street, IPT, C5L100, Los Angeles, CA 90033 USA; 3 Department of Surgery, University of Insubria, Milan, Italy; 4 Thoracic Surgery and Lung Transplant Unit, Fondazione IRCCS Ca’ Granda Ospedale Maggiore Policlinico, University of Milan, Via Francesco Sforza, 35 Milan, Italy

**Keywords:** Esophagectomy, Gastrectomy, Hiatus hernia, Trans-diaphragmatic herniation, Outcomes

## Abstract

**Introduction:**

The anatomy of the esophageal hiatus is altered during esophagogastric surgery with an increased risk of postoperative hiatus hernia (HH). The purpose of this article was to examine the current evidence on the surgical management and outcomes associated with HH after esophagogastric surgery for cancer.

**Materials and methods:**

Systematic review and meta-analysis. Web of Science, PubMed, and EMBASE data sets were consulted.

**Results:**

Twenty-seven studies were included for a total of 404 patients requiring surgical treatment for HH after esophagogastric surgery. The age of the patients ranged from 35 to 85 years, and the majority were males (82.3%). Abdominal pain, nausea/vomiting, and dyspnea were the commonly reported symptoms. An emergency repair was required in 51.5%, while a minimally invasive repair was performed in 48.5%. Simple suture cruroplasty and mesh reinforced repair were performed in 65% and 35% of patients, respectively. The duration between the index procedure and HH repair ranged from 3 to 144 months, with the majority (67%) occurring within 24 months. The estimated pooled prevalence rates of pulmonary complications, anastomotic leak, overall morbidity, and mortality were 14.1% (95% CI = 8.0–22.0%), 1.4% (95% CI = 0.8–2.2%), 35% (95% CI = 20.0–54.0%), and 5.0% (95% CI = 3.0–8.0%), respectively. The postoperative follow-up ranged from 1 to 110 months (mean = 24) and the pooled prevalence of HH recurrence was 16% (95% CI = 13.0–21.6%).

**Conclusions:**

Current evidence reporting data for HH after esophagogastric surgery is narrow. The overall postoperative pulmonary complications, overall morbidity, and mortality are 14%, 35%, and 5%, respectively. Additional studies are required to define indications and treatment algorithm and evaluate the best technique for crural repair at the index operation in an attempt to minimize the risk of HH.

## Introduction

Esophagogastric surgery is the cornerstone for esophageal and gastric cancer. These consist of a demolitive stage with en bloc esophageal or gastric dissection, lymphadenectomy, and restoration of the alimentary tract [1–3]. After the procedure, the normal anatomy around the hiatus can be altered due to surgical maneuvers causing inadvertent injuries to the crural muscular fibers. Therefore, there is an increased risk of postoperative hiatus hernia (HH) with herniation of the intra-abdominal viscera. The incidence of HH after esophagogastric surgery is not well described. A recent systematic review reports a postoperative HH incidence of 2.6% up to 32-month follow-up; however, because of the limited follow-up and the selection bias for some studies reporting data for only HH requiring surgical management, the actual incidence might be underestimated [4].

HH after esophagogastric surgery can cause severe life-threatening complications and critical illnesses in a high percentage of the patients [5, 6]. These patients with HH often present with acute symptoms onset related to strangulation, necrosis, and perforation of the herniated viscera. Symptomatic HH mandates a surgical intervention, including reduction of the herniated intra-abdominal viscera and concomitant cruroplasty to reduce the risk of future recurrences [7]. Current knowledge about incidence, risk factors, and related outcomes is only supported by retrospective studies. Therefore, contemporary evidence about presenting symptoms, timing for surgical repair, treatment strategies, related complications, recurrence, and postoperative mortality is puzzled and unsettled.

The aim of this systematic review and meta-analysis was to examine the current literature on the surgical management and outcomes associated with HH after esophagogastric surgery for cancer.

## Materials and methods

### Search strategy

A systematic review was performed according to the guidelines from the Preferred Reporting Items for Systematic Reviews and Meta-analyses checklist (PRISMA) [8] and Meta-analyses of Observation Studies in Epidemiology (https://www.editorialmanager.com/jognn/account/MOOSE.pdf). The literature search ended on March 15, 2021. An institutional review board approval was not required. The literature search was conducted independently by three authors (AA, GB, and FL) to identify the English-written published series on hiatus hernia after esophagectomy or gastrectomy for cancer. Web of Science, PubMed, and EMBASE data sets were consulted matching the terms “hiatus hernia,” “esophagectomy,” “esophageal resection,” “gastrectomy,” and “gastric resection,” with “AND” and “OR.” The references of each article were assessed to complete the research [9].

### Inclusion and exclusion criteria

Inclusion criteria are as follows: (a) articles reporting outcomes for hiatus hernia after esophagectomy or gastrectomy for cancer; (b) English written; (c) papers with the longest follow-up or the largest sample size in case of articles published by the same study group or based on the same dataset. Exclusion criteria are as follows: (a) not English written; (b) articles not reporting any of the a priori defined primary outcomes; (c) articles describing outcomes for distal gastrectomy or atypical gastric resection; (d) case series and case reports with <5 patients.

### Data extraction

Three authors (FL, CL, and AA) independently extracted data from eligible studies. Data extracted included study characteristics (first author name, year, and journal of publication), number of patients included in the series, time frame, clinical and demographic characteristics of patients’ population, type of surgical procedure, and postoperative outcomes. Disagreements between authors were resolved by consensus; if no agreement could be reached, a fourth senior author (DB) made the decision.

### Quality assessment

The quality of observational studies was independently assessed by three authors (AA, FL, and CL) with the Risk of Bias In Non-Randomized Studies (ROBINS-I) instrument [10]. Confounding, selection, classification, intervention, missing data, outcomes measurement, and reporting bias were considered. Each domain was estimated with “yes,” “probably yes,” “probably no,” or “no,” and studies were categorized as having low, moderate, serious, or critical risk of bias.

### Outcomes

Primary outcomes include the following: overall complications and hospital mortality. Secondary outcomes are as follows: anastomotic leak, pulmonary complications, cardiovascular complications, conversion to open, reoperation, hospital length of stay (HLOS), and hernia recurrence.

### Statistical analysis

We performed a random-effects frequentist meta-analysis. Binary outcomes were pooled using generalized linear mixed models with logit transformation [11, 12]. The maximum likelihood estimator was used to estimate the between-study variance (τ^2^) and the nonparametric bootstrap was used to calculate its bias-corrected and 95% confidence interval. The inverse-variance weighted random-effects frequentist meta-analysis was performed by conventional methods using DerSimonian–Laird estimator for estimate between-study variance (τ^2^) [13]. Clopper–Pearson 95% confidence interval for an individual was computed [14]. Statistical heterogeneity was evaluated (I^2^ index): value of 25% or smaller was defined as low heterogeneity, value between 50 and 75% as moderate heterogeneity, and 75% or larger as high heterogeneity [15, 16]. Small study and publication bias effects were assessed by trim and fill funnel plot visual inspection and Egger tests [17–19]. The prediction interval for the treatment effect of a new study is calculated according to Borenstein et al. [20]. As the sample size is not the same in all studies, we gradually removed a small sample size to perform a sensitivity analysis to assess stability of results. Two-sided p-values were considered statistically significant when <0.05. All analyses and graphical representations were carried out using R version 3.2.2 software [21].

## Results

### Systematic review

**Fig. 1:**
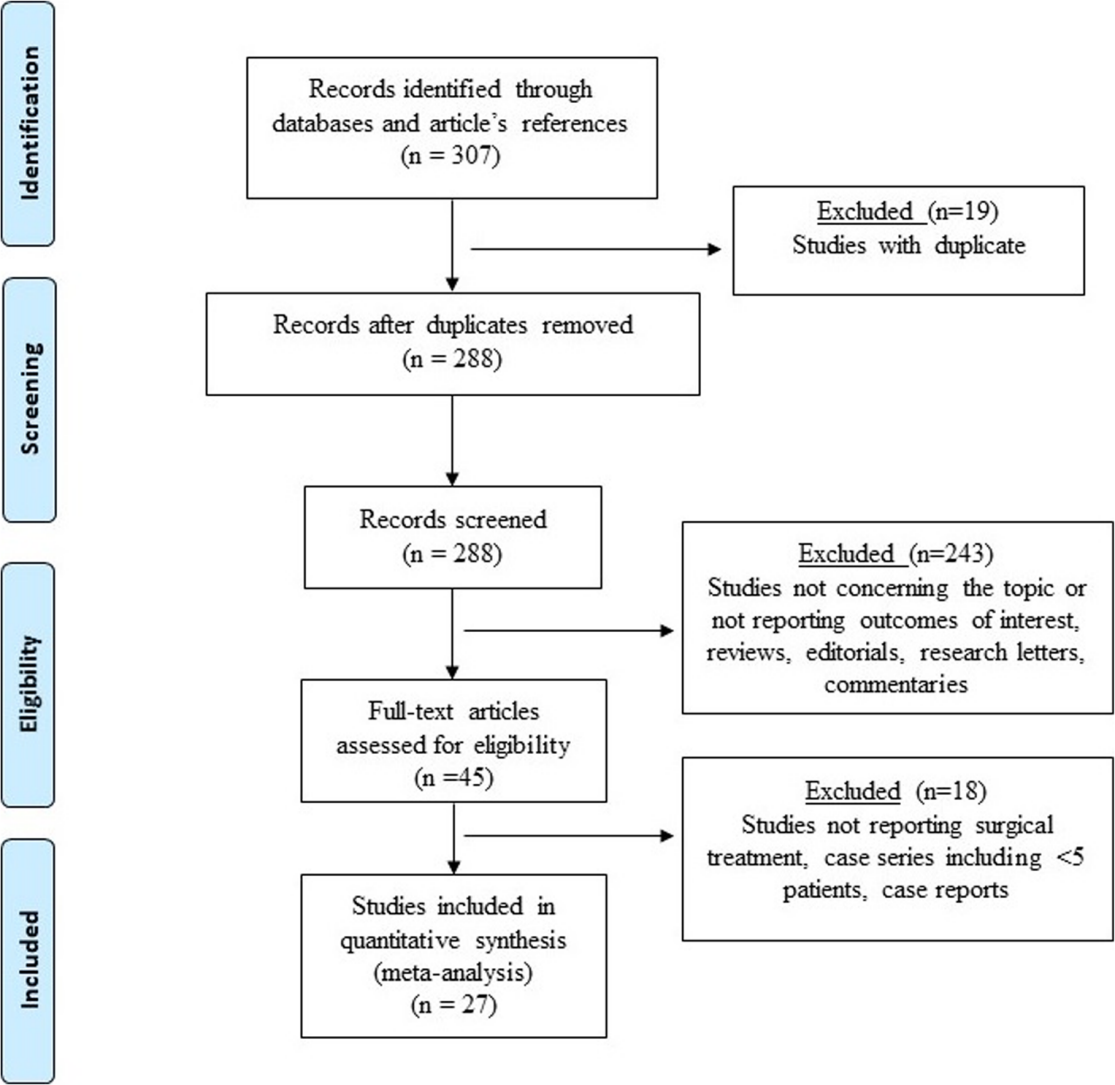
The Preferred Reporting Items for Systematic Reviews and Meta-Analysis checklist (PRISMA) diagram

Twenty-seven studies published between 1999 and 2021 met the inclusion criteria (Fig. 1). Overall, 23 studies reported data for the total number of surgical procedures performed for esophageal gastric cancer (18,125 patients). The prevalence of post-esophagogastric surgery HH was 3.18%, while the prevalence of HH requiring surgical treatment was 2.01%. Overall, 404 patients underwent surgical treatment for HH and were included in the final quantitative analysis. The sample size of the individual studies ranged from 5 to 43. All reports were observational cohort studies. Fourteen studies were classified with moderate risk of bias, while thirteen had serious risk of bias at quality assessment (Supplementary Table 1). Patient demographics, clinical, and operative variables are shown in Table 1. The age of the included patients ranged from 35 to 85 years, the majority were males (82.3%), and the preoperative body mass index (BMI) was reported in 10 articles. Overall, 21 studies (289 patients) described the surgical approach for the index operation: 57.0% had a hybrid or totally minimally invasive esophagectomy, 21.2% open esophagectomy, 15.2% transhiatal esophagectomy, and 6.6% total gastrectomy. The incidence of HH after minimally invasive procedures ranged from 0.0 to 22.1%, while it ranged from 0.0 to 12.3% following open surgery. The pooled incidence of HH was higher after minimally invasive procedures (5.3%; 95% CI = 2.9–6.7) compared to open surgery (1.5%; 95% CI = 0.4–2.2).

**Table 1:** Demographic, clinical, and operative data. *Ret*, retrospective; *nr*, not reported; *BMI*, body mass index. Data are reported as numbers, mean ± standard deviation, and median (range)

Author, year, country	Study design	Period	Total no. of patients	No. pts surgical repair	M/F	Age (y)	BMI (kg/m^2^)	Neoadjuvant therapy	Elapsed time from index operation (mos)	Elective/emergency repair
van Sandick 1999, Netherlands [22]	Ret	1993–1997	219	6	5/1	nr	nr	nr	nr	6/0
Vallbohmer 2007, Germany [23]	Ret	1997–2007	355	7	7/0	50 ± 8	nr	7	8 ± 7.4	2/5
Kent 2008, USA [24]	Ret	1995–2007	1075	22	nr	nr	nr	nr	32 (46 m–7 y)	18/4
Sutherland 2010, USA [25]	Ret	2007–2009	36	7	5/2	67.4 ± 8	77.8 ± 18.3	nr	nr	1/6
Price 2011, USA [26]	Ret	1988–2009	2182	15	14/1	61 ± 10.5	nr	10	21 (3 d–12 y)	13/2
Erkmen 2013, USA [27]	Ret	nr	nr	5	4/1	35–85	nr	4	21.4 ± 11.3	0/5
Ganeshan 2013, USA [28]	Ret	2001–2007	440	9	8/1	56 ± 14	nr	nr	24 (1.5–108)	4/5
Bronson 2014, USA [29]	Ret	2003–2011	118	9	6/3	60.4 ± 14	27.5 ± 7.2	6	13.7 ± 13.4	9/0
Messenger 2015, UK [30]	Ret	1996–2012	273	11	9/2	59 ± 6.9	nr	nr	Y	6/5
Benjamin 2015, USA [31]	Ret	2006–2013	120	5	4/1	58 ± 9.5	nr	5	3.4 ± 11	4/1
Kanamori 2015, Japan [32]	Ret	2010–2014	150	5	5/0	64.8 ± 11	20.1 ± 1.9	1	4.7 ± 3.3	1/4
Severino 2015, France [33]	Ret	2000–2013	390	32	25/7	60 ± 9.7	nr	24	10 (3 d–54 m)	26/6
Narayanan 2015, USA [34]	Ret	2000–2013	199	9	7/2	59.5 ± 9.6	28.4	8	2.4 y	6/3
Crespin 2016, USA [35]	Ret	2004–2013	192	7	6/1	62.1 ± 8.5	25.9 ± 5.9	nr	7.5 ± 24.2	5/2
Matthews 2016, UK [36]	Ret	2001–2015	631	31	31/0	64 ± 9.7	nr	nr	nr	11/20
Andreou 2017, Germany [37]	Ret	2005–2012	471	13	13/0	63 ± 9.7	27 ± 3	11	15 ± 14	5/8
Brenkman 2017, Netherlands [4]	Ret	2000–2014	657	26	25/1	62.8 ± 8.1	24.7 ± 3.5	33	20 (0–101)	31/14
Gooszen 2018, Netherlands [38]	Ret	2005–2016	851	21	15/7	62 ± 8.5	24 ± 3.7	19	5.7 ± 8.5	6/15
Gust 2019, France [7]	Ret	200–2016	6608	43	38/5	59 ± 11	nr	43	nr	43/0
Takeda 2019, Brazil [39]	Ret	2009–2019	328	8	5/3	61.6 ± 5.7	nr	nr	22.5 ± 9.4	nr
Gong 2019, Korea [40]	Ret	2011–2017	490	8	3/5	49.3 ± 9.3	nr	nr	7.3 ± 2.3	0/8
Hanna 2019, USA [6]	Ret	2011–2017	258	17	14/33	62.4 ± 5.4	nr	62	31	9/8
Urabe 2019, Japan [41]	Ret	1985–2013	1361	5	4/1	68.2 ± 3.7	nr	nr	78.1 ± 46.5	1/4
Fuchs 2020, Germany [42]	Ret	2003–2017	nr	39	30/9	60 ± 11.7	23 ± 4	36	8.6 ± 12.1	17/22
Lubbers 2021, Netherlands [43]	Ret	2011–2018	307	8	7/1	62 ± 2.2	25.9 ± 1.5	8	9	0/8
Puccetti 2021, Italy [44]	Ret	1997–2018	414	22	21/1	62 ± 9.7	24.5 ± 3.3	16	8	6/16
Oppelt 2021, Germany [45]	Ret	1998–2018	nr	22	16/6	66 (26–81)	21.6 (15.8–34.7)	nr	12.2 ± 36	12/10

Seventeen studies reported the details of the surgical technique for crural dissection at the index operation. The crural or diaphragmatic opening was not routinely performed, and the decision was left to the operating surgeon’s preference. Partial or complete division of the right/left crus or dissection of the anterior aspect of the diaphragm was described and reported heterogeneously. Similarly, various techniques for hiatus closure at the index procedure were described in 14 studies (leaving the hiatus open, systematic hiatus closure with interrupted sutures, or simple closure reinforced with pledgets). Finally, 11 studies described the technique for diaphragmatic gastric tube fixation. Again, the reported techniques were variable (i.e., “a la demande” interrupted stitches, cardinal interrupted stitches at the 9, 12, and 3 o’clock, continuous suture).

Preoperative symptoms related to HH were reported in 21 studies (n = 329): commonly reported symptoms were abdominal pain (54.7%), nausea/vomiting (33.4%), and dyspnea (24%), while 15.5% were asymptomatic. Various indications for surgical repair of HH were reported, dependent on surgeon preference and hospital inclination. The majority of patients had involvement of the left hemithorax (89.1%), while 3.4% had bilateral visceral herniation. The most commonly reported herniated organ was the colon (51%), followed by combined colon–jejunum (31%) and small bowel (16%). The timing from the index procedure to diagnosis and surgical repair was specified in 19 studies and ranged from 3 to 144 months. The majority of patients (67%) underwent surgical treatment within 24 months from the index cancer surgery. An emergency repair was performed in 51.5% of patients for incarceration or strangulation of the herniated viscera. Minimally invasive laparoscopic repair was successfully performed in 48.5% of patients. After reducing the herniated organs, simple suture cruroplasty was performed in 64.6%, while mesh reinforced repair was necessary in 35.4% of patients. Other techniques such as pexy (colopexy, gastropexy, omentopexy) and crural gastric conduit fixation were reported in a minority of patients. Bowel resection was necessary for 6.9%. The postoperative follow-up was reported in 15 studies and ranged from 1 to 110 months (mean = 23.7).

### Meta-analysis

#### Primary outcomes

**Fig. 2:**
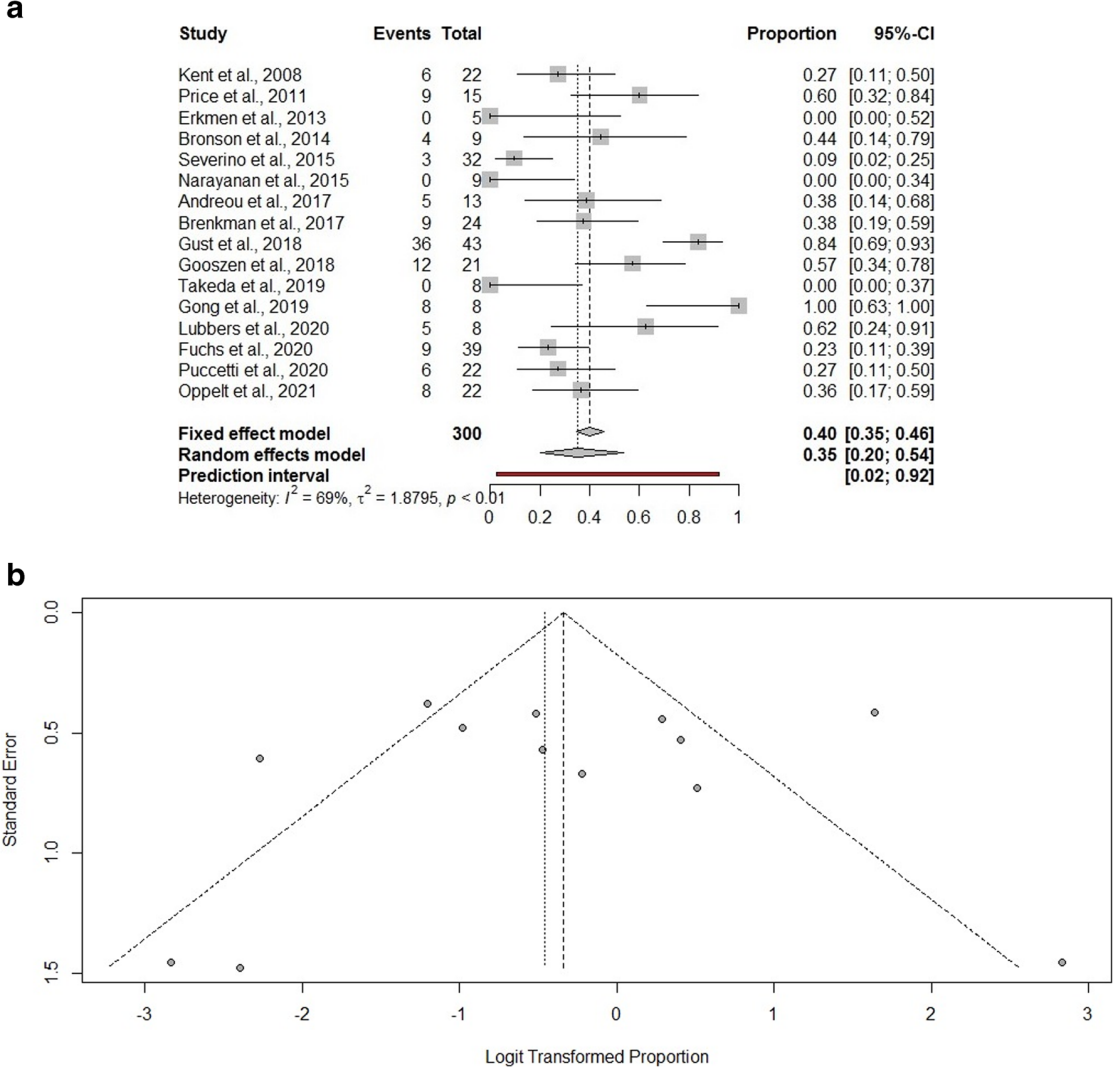
Forest plot (**A**) and funnel plot (**B**) for overall complications

**Fig 3:**
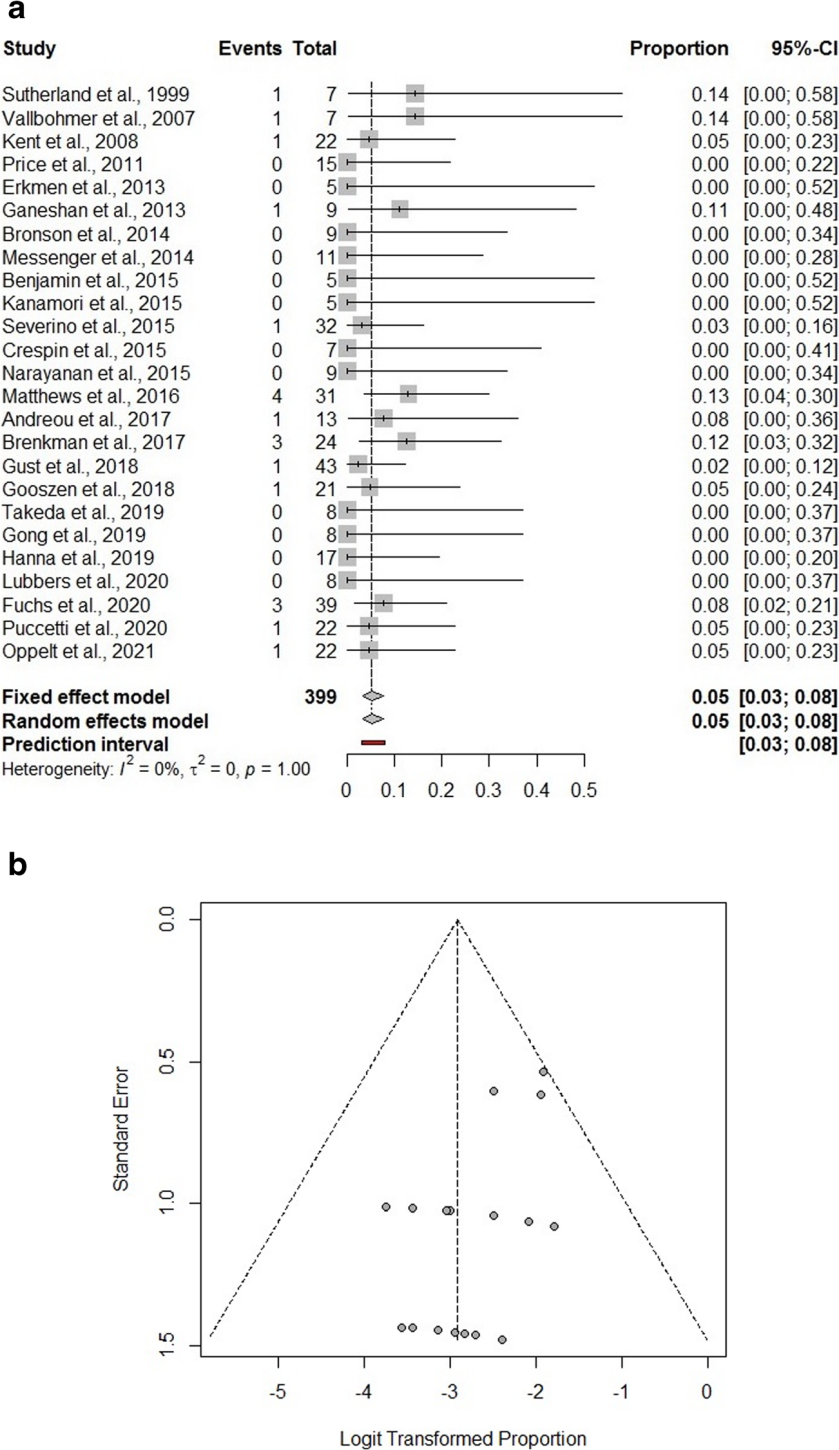
Forest plot (**A**) and funnel plot (**B**) for in-hospital mortality

In addition to a systematic review, we performed a frequentist meta-analysis. Considering random-effects model, the estimated pooled prevalence of overall morbidity (16 studies, 300 patients) is 35% (95% CI = 20.0–54.0%) (Fig. 2A–B). The prediction lower and upper limits are 2.0% and 92.0%, respectively. The heterogeneity index is high (I^2^ = 69%, 95% CI = 59.6–95.7%; p < 0.01). The sensitivity analysis shows the robustness of the results. The estimated pooled prevalence of hospital mortality (25 studies, 399 patients) is 5.0% (95% CI = 3.0–8.0%) (Fig. 3A–B). The prediction lower and upper limits are 3.0% and 8.0%, respectively. The heterogeneity index is 0 (I^2^ = 0.0%, 95% CI = 0.0–8.4%; p = 0.54). The sensitivity analysis shows the robustness of the results.

#### Secondary outcomes

The estimated pooled prevalence of anastomotic leak (17 studies, 335 patients) is 1.4% (95% CI = 0.8–2.2%). The prediction lower and upper limits are 0.3% and 4.3%, respectively. The heterogeneity index is moderate (I^2^ = 31%). The sensitivity analysis shows that omitting the study by Gust et al., the heterogeneity decreases to 0.0%. The pooled prevalence of cardiovascular complication (14 studies, 283 patients) is 9.0% (95% CI = 4.1–16.0%) with a moderate heterogeneity (I^2^ = 68%). The pooled prevalence of pulmonary complication (18 studies, 344 patients) is 14.1% (95% CI = 8.0–22.0%) with a moderate heterogeneity (I^2^ = 27%). The pooled prevalence rates of conversion to open procedure (18 studies) and reoperation (22 studies) are 9.2% (95% CI = 4.0–21.0%) and 12.0% (95% CI = 8.0–17.3%), respectively. The estimated pooled mean HLOS (16 studies) is 15.9 days (I^2^ = 93%). The pooled prevalence of recurrence after HH repair (22 studies) is 16.0% (I^2^ = 0.0%) (Table 2).

**Table 2:** Secondary outcomes values are expressed as pooled proportions and 95% confidence intervals (95% CI). *I*^2^, heterogeneity; *HLOS*, hospital length of stay

Outcomes	Proportion (95% CI)	I^2^ (95% CI)	No. of studies
Cardiovascular complications	9.0% (4.1–16.0%)	68% (26–95%)	14
Pulmonary complications	14.1% (8.0–22.0%)	27% (5.3–49.0%)	18
Anastomotic leak	1.4% (0.8–2.2%)	31% (0.0–53.1%)	17
Reoperation	12.0% (8.0–17.2%)	0.0% (0.0–78%)	22
Conversion to open	9.2% (4.5–21.0%)	0.0% (0.0–67%)	18
Recurrence	16.0% (13–22.6%)	0.0% (0.0–26.1%)	22
HLOS (days)	15.93 (10.4–21.4%)	93% (76.2–99.4%)	16

## Discussion

Our systematic review suggests that evidence reporting data for hiatus hernia after esophagogastric surgery is scarce and only supported by retrospective studies. In the present analysis, the incidence of post-esophagectomy/gastrectomy HH is about 3%, while up to 2% of patients may require surgical treatment. Postoperative overall complications rate is up to 40%, while anastomotic leak and pulmonary complications are commonly reported. The postoperative hospital mortality is estimated up to 5%.

There is limited experience with trans-diaphragmatic visceral herniation after esophagogastric surgery even at high-volume centers, and current evidence is mainly ascribed to observational studies. The actual prevalence of transhiatal herniation after esophagogastric surgery is a matter of discussion, with previous studies reporting a wide range of incidence. This is likely due to the various definitions of diaphragmatic hernia and the heterogeneous inclusion criteria. All these issues, in conjunction with the retrospective design of studies, lack of adherence to standardized follow-up schedules, routine imaging, threshold for clinical suspicion, and presence of mild symptoms, may be responsible for the real prevalence of underreporting and underestimation [5, 36]. For example, Brenkman et al. reported a HH incidence of 7% after esophagectomy. This percentage was probably underestimated because up to 25% of patients that underwent esophagectomy did not undergo a computed tomography scan [3]. On the other hand, Ganeshan et al. reported a higher percentage of HH (up to 15%) in their study, including both symptomatic and asymptomatic patients [28]. A recently published analysis reported a mean HH incidence of 2.6% during the postoperative follow-up to 32 months [31]. Similarly, in the present analysis, the incidence of HH has been estimated at around 3%, with almost 2% of subjects requiring surgical repair. Although transhiatal herniation may develop in both open and minimally invasive procedures, we observed a trend toward a higher incidence of HH after minimally invasive surgery (5.3% vs. 1.5%). This result is in line with previous studies reporting a significantly higher incidence of HH after minimally invasive surgery [5]. It has been proposed that decreased postoperative intra-abdominal adhesions and a larger size hiatal defect created by minimally invasive approaches are associated with a higher incidence of HH [24, 30]. In contrast, other studies reported the use of a minimally invasive approach was not identified as an independent predictor for HH in the multivariate analysis [4]. Therefore, whether a minimally invasive approach would increase the risk of HH is still unclear. Symptomatic HH may occur both in the short- and long-term follow-up and will presumably become an increasing problem as cancer survival improves. In the present study, the timing of presentation ranged from 3 to 144 months, with the majority of patients (67%) being diagnosed within 24 months from the index cancer procedure. This result supports the data reported by Brenkman et al. that described a higher risk of HH within the first 2 years from the index procedure [4].

The pathophysiology of diaphragmatic visceral herniation after esophagogastric surgery has been extensively discussed. As reported in previous studies, pre-existing hiatal hernia, surgical widening of the hiatus, accidental pleural laceration, advanced tumor stage with partial crural en bloc resection, and high abdominal–thoracic pressure gradient are risk factors. Additionally, lower BMI (<25 kg/m^2^) or excessive weight loss after index surgery, diabetes, total number of harvested lymph nodes, transhiatal approach, and neoadjuvant therapy have been advocated as further risks [22–45]. In an attempt to restore the hiatus function and reduce the incidence of HH, several technical features have been described such as direct closure of the diaphragmatic defect, crural mesh reinforcement, fixation of the conduit to the crura, and omentopexy at the index operation. However, data are sparse and conclusive evidence about the most appropriate technique for HH prevention is lacking. In the present systematic review, only 14 studies described the technique for crural reconstruction at the index procedure. Therefore, any attempt at quantitative analysis was not possible because data were heterogeneous and outcomes reported as aggregated. Further studies are necessary to assess the best technique for crural repair in attempt to minimize the risk of HH [46].

Abdominal pain, nausea/vomiting, and dyspnea were commonly reported. These symptoms should be always monitored carefully in patients with previous esophagogastric surgery as these can be signs of mild HH. Therefore, it is recommended to have a high index of suspicion for a prompt diagnosis [4, 6, 47]. Dysphagia and weight loss were less frequently reported; however, tumor recurrence should always be excluded in these patients. While symptomatic HH represents an absolute indication for surgical intervention, asymptomatic or mild symptomatic patients pose a dilemma for surgeons. A conservative wait-and-see approach in patients without symptoms has been proposed by some authors. In contrast, others recommended an elective surgical repair even in asymptomatic patients because of the subtle risk of hernia enlargement and possible evolution through complication [6, 26, 42]. Current evidence is narrow and heterogeneous, while a robust indication for conservative vs. operative treatment in asymptomatic or mild symptomatic patients with small- to medium-size hernia is still unsolved. Therefore, additional studies are required to better define a precise treatment algorithm in these patients. The decision should be patient tailored while “pros” and “cons” should be balanced and individualized according to prognosis, underlying diseases, and patient wishes.

Emergent or urgent repair may be required in the case of severe HH-related complications such as incarceration, ischemic bowel complications, strangulation, or bowel perforation [28, 48]. In the present study, about 51% of patients underwent urgent/emergent repair and bowel resection was performed in about 7%. To date, different types of operative strategies have been described. Both open and laparoscopic approaches have been advocated as safe and feasible options. However, surgeons should be prepared to undertake a thoracic approach in case of severe thoracic adhesions that prevent the reduction of the prolapsed contents into the abdominal cavity. During the operation, the viability of the herniated viscera should be evaluated and closure of the diaphragmatic defect is recommended to possibly reduce the risk of HH recurrence. While simple suture cruroplasty was performed in 65%, mesh reinforced repair was adopted in 35% of patients. Currently, a definitive indication about the most appropriate technique for crural repair is lacking and further studies are warranted to deeply assess this issue.

Transhiatal herniation after esophagectomy and gastrectomy has been shown to be associated with non-negligible postoperative complications and mortality. The present quantitative analysis showed that the postoperative morbidity and mortality RR are 35% (95% CI = 20.0–54.0%) and 5.0% (95% CI = 3.0–8.0%), respectively. While the heterogeneity for overall morbidity is high, thus probably reflecting different articles reporting and the definition of postoperative complications across studies, the related heterogeneity for postoperative mortality was 0.0%, thus adding robustness to the result. These data seem similar to previous studies reporting postoperative morbidity and mortality rates up to 45% and 10% [22–45]. It has been reported that postoperative mortality may be higher in patients that presented with acute symptoms and required an emergent operation; however, a subgroup analysis including emergency cases was not feasible because the data were reported as aggregated. Interestingly, pulmonary complications (RR = 14.1%, 95% CI = 8.0–22.0%) were the most commonly observed postoperative complications. Therefore, surgeons should be aware of these life-threatening complications that require prompt diagnostic workup and management. The pooled mean hospital length of stay was 15.9 days (95% CI = 10.1–21.5) with high related heterogeneity (I^2^ = 93%). This may be explained by several factors such as patients’ age, comorbidities, preoperative BMI, surgical technique, need for visceral resection, hospital volume, and surgeons’ expertise.

The postoperative follow-up ranged from 1 to 110 months. The estimated RR for recurrence was 16% (95% CI). The related heterogeneity was high, thus probably reflecting the significant variability within surgical techniques for crural reconstruction, operating surgeon’s expertise, and patients’ comorbidities. Therefore, evidence to support one technique over another for crural repair and hiatus approximation is lacking, while future studies should focus on this issue to possibly minimize such complication.

We acknowledge that this review does have some limitations related to possible publication bias due to the exclusion of non-English articles, heterogeneity of some of the studies included, and retrospective nature of the included series. As a result of the retrospective design of included studies, lack of routine imaging, and short follow-up, the exact incidence of HH might be underreported. Various techniques for crural/diaphragmatic dissection, crural approximation, and gastric tube fixation at the index operation were described. In addition, a specific surgical approach was usually chosen for each case based on the operating surgeon’s preference and may represent a selection bias. Finally, the limited patient cohort may constitute a further limitation. However, it should be noted that HH after esophagogastric surgery is a relatively rare complication with few publications and limited patients’ cohorts. Therefore, this meta-analysis aims to plea for further qualitative and standardized studies in order to codify the best surgical technique for hiatus closure at the index procedure, to further assess a precise indication about asymptomatic patients (conservative vs. surgery), and find out a universally accepted approach and treatment algorithm for such cases. Finally, future studies should be focused on the identification of predictive factors for HH in high-risk patients where preventive measures can be attempted and closer postoperative follow-up applied.

## Conclusions

Current evidence reporting data for HH after esophagogastric surgery is limited and only supported by retrospective studies. While the actual incidence remains unknown, the present study demonstrates that the incidence of post-esophagectomy/gastrectomy HH is 3%, while surgical management is necessary in about 2% of patients. The overall postoperative complications rate may occur in up to 35% of patients with pulmonary complications being commonly reported. The postoperative mortality is estimated up to 5%. Additional studies are required to define indications and treatment algorithm and evaluate the best technique for crural repair at the index operation in an attempt to minimize the risk of HH.
